# Supplementary material for: Identification of New CTX Analogues in Fish from the Madeira and Selvagens Archipelagos by Neuro-2a CBA and LC-HRMS
Source: Mar Drugs. 2022 Mar 29;20(4):236. doi: 10.3390/md20040236 (PMC9031360; doi:10.3390/md20040236)
Supplement: Supplementary file 1 [file marinedrugs-20-00236-s001.zip › marinedrugs-1642858 supp table highlight.pdf]

Table S1. List of GPS coordinates and depth of capture of individuals captured between October 2013 and December 2014 in the coasts of Madeira Archipelago including Madeira, Desertas and Selvagens Islands included in Table 1.

Code	Species	Location	GPS coordinates	Depth of capture (m)
1	*Mycteroperca fusca*	Desertas Islands	-	-
2	*Chelon labrosus*	Desertas Islands	-	-
6	*Bodianus scrofa*	Selvagens Grande	-	-
13	*Kyphosus sectatrix*	Selvagens Grande	-	-
14	*Balistes capriscus*	Selvagens Grande	-	-
25	*Gymnothorax unicolor*	Selvagens Grande	-	-
26	*G. unicolor*	Selvagens Grande	-	-
27	*G. unicolor*	Selvagens Grande	-	-
28	*G. unicolor*	Selvagens Grande	-	-
29	*C. labrosus*	Desertas Islands	-	-
31	*Sparisoma* sp.	Desertas Islands	-	-
34	*Muraena augusti*	Desertas Grande	32º31'05,07'' N 16º30'49,50'' W	6
35	*M. augusti*	Desertas Grande	32º30'57,30'' N 16º30'47,60'' W	6
36	*Muraena helena*	Desertas Grande	32º31'05,07'' N 16º30'49,50'' W	6
37	*M. helena*	Desertas Grande	32º31'05,07'' N 16º30'49,50'' W	6
38	*M. helena*	Desertas Grande	32º30'57,30'' N 16º30'47,60'' W	6
39	*M. helena*	Desertas Grande	32º30'57,30'' N 16º30'47,60'' W	6

Table S2. Identification criteria using full scan LC-HRMS analysis in ESI + mode for C-CTX1.

Sample ID	Matrix	Retention time (min)	**C-CTX1 (M, $C_{62}H_{92}O_{19}$, MW 1140.6227)**			
			**$[C\text{-}CTX1+H]^+$ ($C_{62}H_{93}O_{19}$)**			
			**Theoretical values**			
			m/z		**RDBE**	**Ratio M+1/M**
			1141.6305		16.5	0.67
			**Experimental-data**			
			m/z	**Mass accuracy (ppm)**	**RDBE**	**Ratio M+1/M**
25	Liver	7.59	1141.6329	2.0	16.5	0.55
26	Muscle	-	-	-	-	-
26	Liver	7.60	1141.6319	1.2	16.5	0.60
27	Muscle	-	-	-	-	-
27	Liver	7.52	1141.6350	3.9	16.5	0.69
35	Liver	7.65	1141.6334	2.5	16.5	0.68

Table S3. Identification criteria using full scan LC-HRMS analysis in ESI$^+$ mode for putative analogue dihydro-CTX2.

Sample ID	Dihydro-CTX2 (M, $C_{60}H_{84}O_{18}$, MW 1092.5652)					
	Matrix	Retention time (min)	$[CTX2\text{-}2H+Na]^+$ ($C_{60}H_{84}O_{18}Na$)			
			Theoretical values			
			m/z		RDBE	Ratio M+1/M
			1115.5611		18.5	0.67
			Experimental-data			
			m/z	Mass accuracy (ppm)	RDBE	Ratio M+1/M
25	Liver	-	-	-	-	-
26	Muscle	6.65	1115.5609	5.3	18.5	0.58
26	Liver	6.70	1115.5619	6.2	18.5	0.64
27	Muscle	6.63	1115.5614	5.7	18.5	0.65
27	Liver	-	-	-	-	-
35	Liver	6.51	1115.5610	5.4	18.5	0.63

Table S4. Identification criteria using full scan LC-HRMS analysis in ESI$^+$ mode for gambieric acid A.

Sample ID	Gambieric acid A (M, $C_{59}H_{92}O_{16}$, MW 1056.6379)													
	Matrix	Retention time (min)	[Gambieric acid A+H]$^+$ ($C_{59}H_{93}O_{16}$)				[Gambieric acid A+NH$_4$]$^+$ ($C_{59}H_{96}O_{16}N$)				Gambieric acid A+Na]$^+$ ($C_{59}H_{92}O_{16}Na$)			
			Theoretical values											
			m/z		RDBE	Ratio M+1/M	*m/z*		RDBE	Ratio M+1/M	*m/z*		RDBE	Ratio M+1/M
			1057.6458		13.5	0.64	1074.6726		12.5	0.64	1079.6278		13.5	0.64
			Experimental-data											
			m/z	Mass accuracy (ppm)	RDBE	Ratio M+1/M	*m/z*	Mass accuracy (ppm)	RDBE	Ratio M+1/M	*m/z*	Mass accuracy (ppm)	RDBE	Ratio M+1/M
25	Liver	-	-	-	-	-	-	-	-	-	-	-	-	-
26	Muscle	-	-	-	-	-	-	-	-	-	-	-	-	-
26	Liver	-	-	-	-	-	-	-	-	-	-	-	-	-
27	Muscle	3.86	1057.6495	3.5	13.5	0.59	1074.6749	2.4	12.5	0.79	1079.6328	4. 7	13.5	0.61
27	Liver	-	-	-	-	-	-	-	-	-	-	-	-	-
35	Liver	4.00	1057.6458	0.1	13.5	0.59	1074.6722	0.2	12.5	0.51	1079.6282	0.4	13.5	0.60

Table S5. Identification criteria using full scan LC-HRMS analysis in ESI$^+$ mode for putative CTX analogue (C-CTX-1109).

Sample ID	Putative CTX analogue (C-CTX-1109)							
	Matrix	Retention time (min)	[CTX analogue+NH_4]$^+$ m/z 1127.6023			[CTX analogue+Na]$^+$ m/z 1132.5577		
			Theoretical values					
			m/z		Ratio M+1/M	m/z		Ratio M+1/M
			1127.6023		0.64	1132.5577		0.64
			Experimental-data					
			m/z	Mass accuracy (ppm)	Ratio M+1/M	m/z	Mass accuracy (ppm)	Ratio M+1/M
25	Liver	7.14	1127.6099	6.7	0.63	1132.5645	6.0	0.53
26	Muscle	7.03	1127.6092	6.1	0.57	1132.5639	5.5	0.55
26	Liver	7.07	1127.6082	5.2	0.57	1132.5625	4.2	0.66
27	Muscle	7.02	1127.6087	5.6	0.55	-	-	-
27	Liver	7.06	1127.6119	8.5	0.53	1132.5603	2.3	0.58
35	Liver	6.87	1127.6098	6.6	0.53	1132.5572	0.5	0.58
